# Guidelines Are Urgently Needed for the Use of Preprints as a Source of Information

**DOI:** 10.2188/jea.JE20200506

**Published:** 2021-01-05

**Authors:** Kazuki Ide, Hitoshi Koshiba, Philip Hawke, Misao Fujita

**Affiliations:** 1 Uehiro Research Division for iPS Cell Ethics, Center for iPS Cell Research and Application (CiRA), Kyoto University, Kyoto, Japan; 2 The Institute of Natural Sciences, College of Humanities and Sciences, Nihon University, Tokyo, Japan; 3 National Institute of Science and Technology Policy, Tokyo, Japan; 4 School of Pharmaceutical Sciences, University of Shizuoka, Shizuoka, Japan; 5 Institute for the Advanced Study of Human Biology (ASHBi), Kyoto University, Kyoto, Japan

In response to the recent COVID-19 pandemic, preprints of articles on this disease have been uploaded to preprint servers at a remarkable rate. While preprints can certainly serve as a useful source of preliminary information, this information has yet to be peer reviewed, and is therefore at greater risk of error and unreliability. Retraction Watch reports that eleven articles on COVID-19 had been withdrawn from preprint servers up to October 2, 2020.^1^ In this letter, we use quantitative data to examine in greater detail the issue of preprint withdrawal and the risk of using preprints as a source of information. We also suggest guidelines for avoiding this risk.

The increasing popularity of preprint servers clearly demonstrates that making scientific information that has not yet undergone peer review publicly available can be valuable in facilitating the timely discussion of preliminary results. However, errors in this information can not only lead to complications for scientists citing it in their research, but can also cause confusion among medical professionals and government officials using it to manage the emerging pandemic.^2^ For example, an article in *The Lancet Infectious Disease* by Verity et al cited a preprint article posted to the medRxiv server by Yang et al which focused on the epidemiological and clinical situation related to COVID-19 in China.^2^^,^^3^ This preprint paper was later withdrawn, however, forcing Verity et al to announce a correction to their own paper. Yang et al stated in a withdrawal letter that the “methods and the main conclusions in our original analyses remain solid” and that they “will replace it with a more up-to-date version shortly.” However, no such updated version had appeared on medRxiv over eight months later. Significantly, the Yang et al preprint was also cited in a report on COVID-19 published by Imperial College London that was aimed at a general readership and which received substantial media coverage.^4^ Cases such as this reveal that the current handling of preprints is causing confusion and perhaps even misinformation.

**Figure 1.**
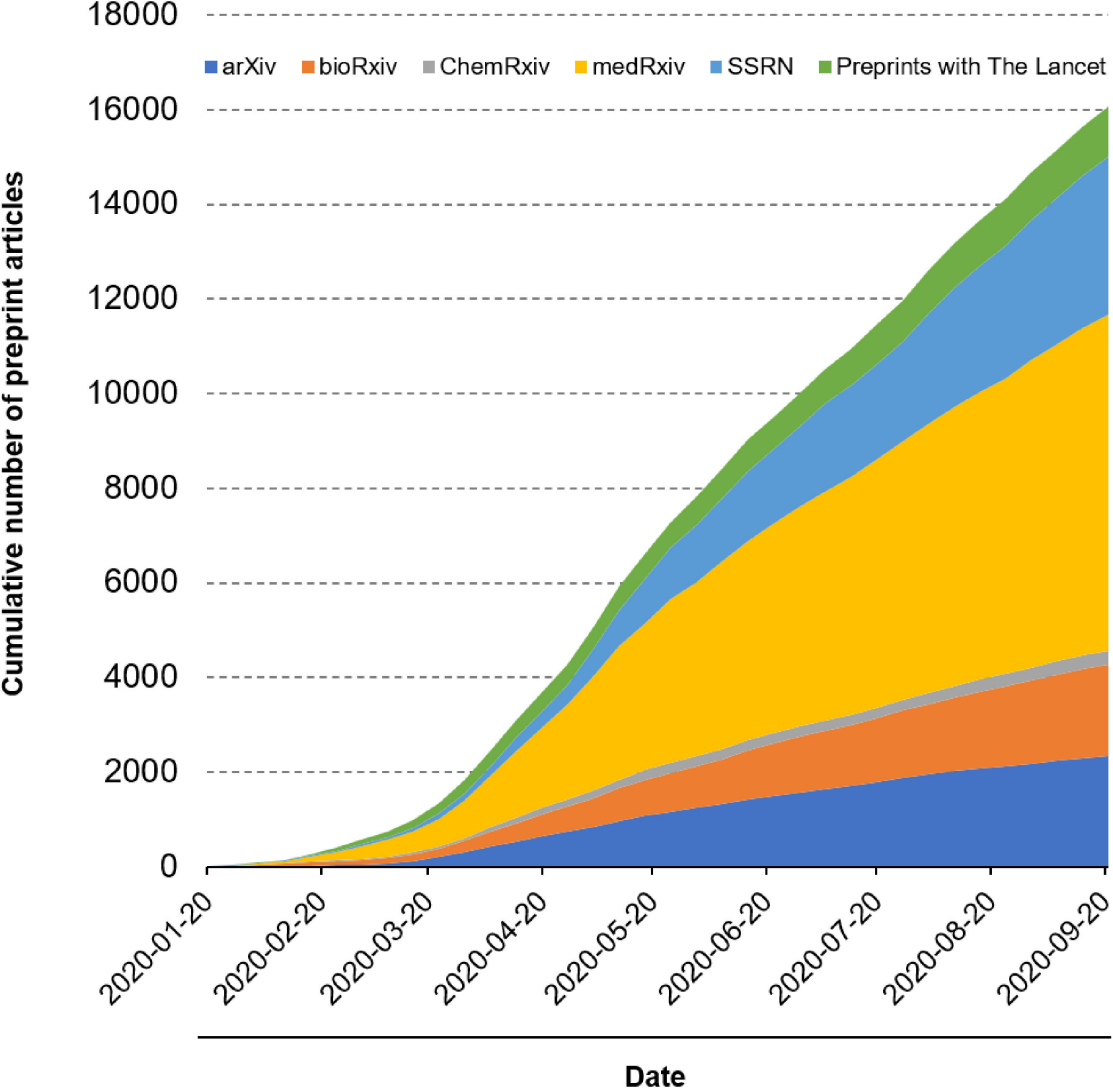
Preprint article publications on COVID-19 over time Cumulative graph showing the number of preprint articles about COVID-19 published on six preprint servers (arXiv, ChemRxiv, medRxiv, bioRxiv, Social Science Research Network (SSRN), and Preprints with The Lancet).

To better understand the problems surrounding preprints, we compiled a database of postings to six major preprint servers over time (arXiv, ChemRxiv, medRxiv, bioRxiv, Social Science Research Network (SSRN), and Preprints with The Lancet), as visualized in Figure 1. We found that 16,066 articles had been posted as of the end of September 2020.

We then searched this database for withdrawals. On medRxiv, the most frequently used preprint server for medical research, six preprints had been withdrawn. Although a notice of withdrawal was clearly stated on the web page for each paper, the reason given for the withdrawal was in the majority of cases quite vague, such as “The authors have withdrawn their manuscript whilst they perform additional experiments to test some of their conclusions further.” To the server’s credit, the previous versions of the papers were still available on the web page for each article. Nevertheless, the PDFs of these previous versions did not have withdrawal notices printed directly on them, an oversight which could easily lead to confusion when copies of the PDF are downloaded. Of greater concern, two studies on the use of ivermectin against COVID-19 posted on SSRN had been withdrawn, and both the papers themselves and the web pages recording their posting history had completely disappeared from the server. No explanation for the withdrawals could be found. The deletion of these papers may be particularly significant as ivermectin has received considerable attention as a potential treatment. These findings also point to the difficulty of using preprints as a source of information.

Several factors contribute to this difficulty. The screening process for posting on preprint servers takes only a few days (2 days for bioRxiv, 4–5 days for medRxiv).^5^ This is much shorter than even the average 60 days required by the new rapid peer review policies instituted by many journals in response to the recent surge in COVID-19-related articles.^5^ In addition, depending on the server, changes in the status of a preprint may not be made clear. In the case of retracted peer-reviewed articles, the standard policy is for the publisher to keep the article in its original location with a retraction notice attached to it. However, in the case of withdrawn preprints, the policy depends on the server. Some servers allow preprints to be simply removed, preventing any follow up of information they contain that has been cited or used for other purposes. Even when a withdrawn paper does remain archived on its web page with a withdrawal notice, the notice may not be printed directly on the previous versions of the paper, and the reason for the withdrawal may not be stated, or stated only vaguely. Furthermore, some servers allow authors to freely update their manuscripts without requiring them to archive previous versions.

Problems such as these highlight the need for best-practice guidelines, both for servers posting preprints and for individuals using them as a source of information. We suggest the following as a starting point. On the server side, a permanent record should be created for each preprint, including an archive of all previous versions. If the preprint is withdrawn, the reason for the withdrawal should be stated clearly and in detail. All previous versions of the paper should be permanently archived, and a withdrawal notice should be clearly printed on the PDF of each version. On the user side, any work which includes information from a preprint should clearly state that the information has not been peer reviewed and so must be treated with due caution.

In 2018, the Committee on Publication Ethics (COPE) published general recommendations related to preprint screening, revisions, and related ethical issues.^6^ However, in the light of the current situation, we think that more detailed guidelines are necessary. We encourage cooperation between COPE and the preprint platforms to establish specific industry-wide standards for the handling of preprints.

In summary, we recognize and emphasize the importance of preprints as a means of ensuring timely discussion. However, as preprints can affect not only other research, but also the understanding of health officials and the public, guidelines on their use as a source of information are urgently needed.
